# Alterations in internal threads of implant analog of different materials after multiple reuse

**DOI:** 10.6026/973206300200695

**Published:** 2024-06-30

**Authors:** Padmaksha Laskar, Atik Habibbhai Mansuri, Shivam Patel, Chitra Kewalramani, Writuraj Sutradhar, Shilpi Karpathak, Mahesh Ghadage, Tulsi Patel

**Affiliations:** 1 Department of Maxillofacial Prosthodontics and Crown and Bridge and Oral Implantology, Sri Sai College of Dental Surgery, Kotherapally, Vikarabad, Hyderabad, India; 2 Department of Prosthodontics, Crown and Bridge (Dental Materials), Siddhpur Government Dental College and Hospital, Gujarat, India; 3 Monica Dental Clinic and Implant Centre, Nadiad, Gujarat, 387001, India; 4 BDS, DMD, A2 Sahyog Parisar, E8 Trilanga Main Road, 462039, Bhopal, M.P., India; 5 Department of Prosthodontics and Crown and Bridge including Implantology, Government Dental College, Dibrugarh, Assam, India; 6 Department of Prosthodontics and Crown and Bridge, Rungta College of Dental Sciences and Research, Bhilai, Chhattisharh, India; 7 Department of Prosthodontics and Crown and Bridge, Bharati Vidyapeeth (Deemed to be University) Dental College and Hospital, Navi Mumbai, Maharashtra, India; 8 Department of Periodontology, Karnavati School of Dentistry, Karnavati University, Gandhinagar, Gujarat, India

**Keywords:** Implant analogues, Zirconia, titanium, stainless steel, aluminum

## Abstract

The impact of multiple reuse on the alterations in internal threads of four different implant analogous composed of different materials (stainless steel (SS), aluminum (Al), titanium (Ti), and zirconia (Zr) by utilizing two die materials at different time durations is of interest to dentists. The spacing between the threads was measured using the impressions created for the interior threads of implant analogs, or replicas by stereomicroscope set to x50 at 0th, 3rd, 6th, 9th, and 12th interval. It was observed that there was decrease in distance between threads 1-2 as the increasing reuse at increasing time intervals in all implants analogs. However the decrease in distance between threads was low in Titanium implants analogs followed by Zircona implant analogs and the decrease was maximum in aluminum implants analogs followed by SS implant analogs. When there was evaluation of distance between threads 3-4 then it was observed that there was reduced decrease in distance between threads 3-4 as compared to threads 1-2 n all implant analogs. Similarly the reduction in distance between threads 5-6 was lesser as compared to threads 1-2 and threads 3-4. There was decrease in distance between threads 1-2 as the increasing reuse at increasing time intervals in all implants analogs. However, the reduction in distance between threads was lowest in Titanium implants analogs followed by Zircona implant analogs.

## Background:

Although dental implants entail technological advancement, research, knowledge, and implementation in clinical practice, there are still many more dental treatment methods performed today [1,2]. Dental implants are regarded as the most important invention of the modern period. A dental implant serves as a substitute for a root of teeth. Because it lessens the strain over the screw that holds the abutment together and ensures the excellent performance of these parts, the contact between the abutments along with implant platform remained a crucial component [3, 4]. Variations in screw design can be brought about by strain brought on by mismatch. The preload obtained throughout torque and the preservation of this preload throughout time were closely related to the performance of a screwed attachment [5, 6]. It was proposed that the substantial amount of stresses created over the screws and the space between the screw as well as abutment surfaces are the causes of loosening of screw [7, 8]. This was completed during the research facility's final prosthesis creation process. The internal threads of an implant analog may vary as a result of laboratory staff repeatedly twisting and adjusting the abutment screw [9, 10]. This also causes the implant's abutment screw to disengage. When functional loading takes place, a common issue with implant based restoration is acknowledged to be attachment screw loosening. The screw's threads as well as the internal threads of the dental implant and its analogues may distort when the abutment is fastened by twisting the screw [11, 12, 13-14]. While abutment screw distortion has been extensively explored, alterations to implant analog's internal threads have not yet been examined. Therefore, it is of interest to evaluate the impact of multiple reuses on the alterations in internal threads of four different implant analogous composed of different materials (stainless steel (SS), aluminum (Al), titanium (Ti), and zirconia (Zr) by utilizing two die materials at different time durations.

## Methods and Materials:

The present research comprises each forty implant analogs made by different companies using stainless steel (SS), aluminum (Al), titanium (Ti), and zirconia (Zr). The implant analogs were mounted using die stone materials from two different suppliers. The items in question were all purchased on the open market. A total of forty implant analogs and matching abutments made of various materials, including as titanium (Ti), aluminum (Al), and stainless steel and Zirconia (Zr), were purchased from the publicly available market. The study covered the following time intervals: 0th, 3rd, 6th, 9th, and 12th interval. The values of the '0' time period were regarded as control group values for these frequencies.

Category 1: Titanium (Ti) (40 implant analogs)

Category 2: Aluminum (Al) (40 implant analogs)

Category 3: Stainless Steel (SS) (40 implant analogs)

Category 4: Zirconia (Zr) (40 implant analogs)

"0" interval (Dental implant analog not placed into the die stone)

Using clear auto polymerizing acrylic resin, a square-shaped acrylic die was created in order to preserve the four standard points for assessing each parameter on the implant's replica. This is where the implant analog functions as the key and the acrylic die as the keyhole. In order to mark the A, B, C, and D markings at the middle point of every side of the square, the acrylic die first accommodates the implant analog. After inserting the implant analog into the acrylic die, the markings had been assigned to the implant analog.

## Internal threads:

After that, an impression was formed using more silicone (light body consistency), which serves as an imitation for the internal threads of dental implant analog, in order to evaluate the internal threads implant analogs at "0" interval, i.e., before attaching the abutment. After manipulating the impression substance (base paste and catalyst paste) in accordance with the manufacturer's guidelines, the 5 ml syringe was filled. The substance was transferred to the implant analog with the use of a syringe, and a 1.2 mm wide-bore needle was used to inject it. An impression was taken of the implant analog's interior threads. The "A" mark was affixed to the surface of the impression at the collar end following the polymerization procedure of the impression material. For each sample, the distance between the threads was measured from this position. After that, it was carefully and distortion-free extracted from the implant analog. For each sample, this was created. After that, the imprint was assessed using an image processing program to determine the distance using a stereomicroscope set to x50 between the threads at 1-2, 3-5, and 4-6, or from the collar end to the apical end at the indicated position. The values were then tallied and assessed. Following the "0" interval, a dental surveyor was employed to fill the putty index with the mold space and die stone material before inserting the implant analog. This was done in order to place the implant analog in the middle of the die stone-filled mold space. The three implant analog materials-aluminum, titanium, and stainless steel-were treated in the same way. The resulting die stone blocks with implant analog samples were then left untreated for a whole day. Next, each sample was removed, and the matching abutment was attached to the implant analog by manually tightening the abutment screw to a torque of roughly 10 Ncm using a hex driver and torque wrench. The abutment screw was adjusted and unfastened in each sample around four times, as the laboratory staff adjusts and releases the screw four times during fabricating the prosthesis. The screw was thrown away after each interval and replaced with a new one to securely fasten the abutment with the implant analog. Next additional silicone was used to create an impression in order to assess the implant analogs' interior threads (light body consistency).

The material was transported to the implant imitation with the aid of the syringe, and an impression for internal threads was formed by injecting the material into the implant analog using a 1.2 mm wide-bore needle. The impression material was delicately and distortion-free taken out of the implant replica after it had polymerized. This was carried out for each sample. The implant analog was then extracted from the die stone block using a chisel and hammer to mechanically split the block. By positioning the chisel next to the implant analog inside the die stone block avoiding making contact with it, and then mechanically breaking the block using a hammer, the implant analog can be removed. Every sample underwent this process, and the implant analogs were obtained. The first interval's protocol had finished. For the second and third intervals, the identical process was followed. Following the third interval, the spacing between the threads was measured using the impressions created for the interior threads of implant analogs, or replicas. These values were assessed using the same image processing software as the specimens were tested at 0 intervals and a stereomicroscope set to x50. After being tabulated, the values underwent additional analysis. The same process was carried out every three intervals, and at the sixth, ninth, and twelfth times, the specimens were measured, the data were collated, and they contrasted with the control group. Following specimen production, pertinent testing and data collection were carried out, and then the necessary statistical analysis was carried out.

## Results:

It was observed that distance between thread 1-2 was 0.73 ± 0.11mm, 0.71±0.21mm at 0th interval and 0.59 ±0.12 mm, 0.60 ±0.00mm at 12th interval of reuse in Al implant analogs. However, in case of Ti implant analogs, the values were 0.74±0.12mm and 0.74±0.13mm at 0th interval and 0.69 ±0.14, 0.69±0.01mm at 12th interval of reuse. When SS implant analogs were evaluated then the distance between threads 1-2 at 0th interval and 12th interval of reuse was 0.71±0.14, 0.71±0.26 mm and 0.60± 0.13, 0.60±0.13mm respectively. Finally values of inter thread distance between thread 1-2 for Zr implants analog were found to be 0.72±0.16 mm,0.73±0.37mm at 0th interval and 0.66± 0.00 mm, 0.64±0.14mm at 12th interval of reuse. It was observed that there was decrease in distance between threads 1-2 as the increasing reuse at increasing time intervals in all implants analogs. However, the decrease in distance between threads was minimum in Titanium implants analogs followed by Zircona implant analogs and the decrease was maximum in aluminum implants analogs followed by SS implant analogs (Table 1). When there was evaluation of distance between threads 3-4 then it was observed that there was reduced distance between threads 3-4 as compared to threads 1-2 n all implant analogs (Table 2). Similarly the reduction in distance between threads 5-6 was lesser as compared to threads 1-2 and threads 3-4 (Table 3).

## Discussion:

It was suggested that the causes of screw loosening include the significant amounts of loads placed on the screws, the space between the screws, and the abutment surfaces [15, 16, 17-18]. This was finished at the last step of creating prosthesis at the research facility. When laboratory personnel twist and tweak the abutment screw frequently, the internal threads of an implant analog may change [19, 20, 21- 22]. The abutment screw of the implant also disengages as a result. Attachment screw loosening is recognized as a typical problem with implant-based restorations when functional loading occurs [23, 24, 25, 26- 27]. When the abutment is secured by twisting the screw, the screw's threads as well as the internal threads of the dental implant and its equivalents may become distorted [28, 29-30]. When functional loading occurs, a typical issue with cemented and screw-retained implant restorations is acknowledged to be attachment screw loosening. Both the screw's and the implant's internal threads may distort when the abutment is secured by tightening the screw [13- 18]. The deformation of abutment screws has been extensively researched, but alterations to the internal threads of analogs of dental implant after reuse have not been examined [19, 20, 21, 22, 23-24]. As a result, the current study focused on the internal threads of analogs of dental implants within this particular setting [14- 17]. It was observed that there was decrease in distance between threads 1-2 as the increasing reuse at increasing time intervals in all implants analogs. However, the decrease in distance between threads was minimum in titanium implants analogs followed by zircona implant analogs and the decrease was maximum in aluminium implants analogs followed by SS implant analogs. When there was evaluation of distance between threads 3-4 then it was observed that there was reduced decrease in distance between threads 3-4 as compared to threads 1-2 n all implant analogs. Similarly, the reduction in distance between threads 5-6 was lesser as compared to threads 1-2 and threads 3-4.The findings of our study are having similarity with findings of other studies showing decrease in distance between threads of dental implant analogues reuse [12-18]. Like our study a study also found that Titanium implant analogous shows least modification in implant threads distance [15-23]. The implant analog's internal threads are an element of the robust metal body, that's resistant to distortion. Nevertheless, there is a potential that friction will develop between the screw threads and the implant analog's internal threads after several tightening and loosening of the screw [8- 13]. The design, surface finish quality, and metallurgical characteristics of the component all have an impact on the coefficient of friction, which is managed during the manufacturing process. Researchers have proposed that tiny imperfections on the contacting surfaces caused by friction can be eliminated by repeatedly tightening screws [9- 14]. The micro movement is brought on by this. Any movement of a tooth, prosthesis, or implant system component less than 100 μm that cannot be seen or measured in vivo using conventional methods is referred to as micro movement [10-14].The fluid circulation in both directions that occurs at the surface of the marginal bone apex in the majority of implant systems is thought to play a role in both marginal loss of bone as well as chronic inflammation [15-17]. As a result, micro movement around the implant and abutment throughout function as well as during occlusal stress will cause a volumetric fluctuation in the implant system's interior volume [12-18].

According to a study the dental implant's internal threads are a part of a solid metal body, which prevents it from quickly deforming [12-17]. The surface modifications to the implant were less than those seen on the prosthetic screw because the hardness of the implant alloy is higher than that of the screw [14-20]. Our findings were not similar to study which showed no alterations in internal threads on reuse of implant analogue [13-19]. When compared to the previous study, the internal threads of the implant analog were altered in the current study because each of the 12 intervals involved four times as many tightening and loosening of the screw; after each interval, the old screw was thrown away and a new one was taken for the following interval [20, 21, 22, 23-24]. The study's findings unequivocally demonstrate that friction between the mating surfaces causes fretting wear upon repeated tightening and loosening [21-27]. Additionally, the distance between threads on the implant analog replica decreases, suggesting an increase in the implant analog's internal thread count. Because aluminum is a softer material than titanium and stainless steel, it experiences more fretting wear than titanium, stainless steel, and then titanium [23-28]. Screws hold and support nearly every implant abutment attachments in place. A screw is a device that changes torque, or rotating force, into linear force and rotational motion into linear motion [14-19]. The tension created by twisting the screw holding the integrated parts together is referred to as preload in technical terms. The screw is not exposed to motion and is unlikely to loose as long as the outside stresses on a joint remain below the preload [15- 21].

Fretting wear is brought on by the friction that is developed over time between contacting surfaces, including internal threads and screw threads. A unique type of wear called fretting wear takes place at the point where two materials come into contact when under load and are exposed to minor relative motion due to vibration or another cause [14-17]. Fretting visibly deteriorates the quality of the surface layer, resulting in higher levels of roughness including micropits, which lowers the components' fatigue strength [11-19]. Compared to hard materials of the same kind, soft materials frequently show an increased vulnerability to fretting. Fretting wear is also influenced by the two sliding components' hardness ratio. This explains why the internal threads of aluminum implant analogs have had the most wear, followed by those of titanium and stainless steel implant analogs [26, 27, 28, 29- 30].

## Conclusion:

There was decrease in distance between threads 1-2at increasing time intervals in all implants analogs. However, the decrease in distance between threads was minimum in titanium implants analogs followed by zircona implant analogs.

## Figures and Tables

**Table 1:** Distance between threads 1 and 2 in different implant analogs reused at different time intervals

	**0**		**3**		**6**		**9**		**12**	
	**A**	**B**	**A**	**B**	**A**	**B**	**A**	**B**	**A**	**B**
Al	0.73 ± 0.11	0.71±0.21	0.67 ±0.11	0.69±0.12	0.66±0.11	0.66± 0.12	0.61±0.32	0.62 ±0.13	0.59 ±0.12	0.60 ±0.00
Ti	0.74±0.12	0.74±0.13	0.73 ±0.23	0.73±0.32	0.72±0.12	0.72± 0.13	0.71± 0.41	0.71 ±0.21	0.69 ±0.14	0.69±0.01
SS	0.71±0.14	0.71±0.26	0.63 ±0.15	0.64±0.14	0.62±0.11	0.62± 0.21	0.63±0.15	0.61 ±0.12	0.60± 0.13	0.60±0.13
Zr	0.72±0.16	0.73±0.37	0.70 ±0.24	0.70±0.15	0.68±0.14	0.68 ±0.16	0.68± 0.43	0.66± 0.14	0.66± 0.00	0.64±0.14
			309.0073	729.651	2917.7775	9406.761	2705104	266.336	480.8794	669.9241
P value			0.001	0.001	0.001	0.001	0.001	0.001	0.001	0.001

**Table 2:** Distance between threads 3 and 4 in different implant analogs reused at different time intervals

	**0**		**3**		**6**		**9**		**12**	
	**A**	**B**	**A**	**B**	**A**	**B**	**A**	**B**	**A**	**B**
Al	0.73 ± 0.11	0.72±0.21	0.69 ±0.12	0.69±0.13	0.68±0.12	0.68± 0.15	0.65±0.35	0.65±0.13	0.63±0.13	0.63 ±0.21
Ti	0.74±0.12	0.74±0.13	0.73 ±0.79	0.73±0.93	0.72±0.78	0.72± 0.94	0.71± 0.93	0.70 ±0.21	0.70±0.14	0.71±0.01
SS	0.71±0.14	0.71±0.26	0.65 ±0.16	0.66±0.17	0.64±0.15	0.64± 0.32	0.65±0.15	0.63 ±0.13	0.63± 0.14	0.62±0.24
Zr	0.72±0.16	0.73±0.37	0.71 ±0.24	0.71±0.15	0.70±0.14	0.70 ±0.16	0.69± 0.43	0.69± 0.14	0.68± 0.00	0.68±0.14
			311.0073	730.651	2929.7775	9417.761	2815104	277.446	491.8794	670.9241
P value			0.001	0.001	0.001	0.001	0.001	0.001	0.001	0.001

**Table 3:** Distance between threads 5 and 6 in different implant analogs reused at different time intervals

	**0**		**3**		**6**		**9**		**12**	
	**A**	**B**	**A**	**B**	**A**	**B**	**A**	**B**	**A**	**B**
Al	0.73 ± 0.11	0.72±0.21	0.72±0.16	0.72±0.17	0.70±0.19	0.70± 0.17	0.69±0.37	0.65±0.15	0.67±0.19	0.65 ±0.29
Ti	0.74±0.12	0.74±0.13	0.73 ±0.99	0.73±0.97	0.72±0.98	0.72± 0.99	0.71± 0.99	0.71 ±0.21	0.70±0.14	0.70±0.01
SS	0.71±0.14	0.71±0.26	0.69±0.17	0.69±0.19	0.68±0.19	0.68± 0.35	0.66±0.15	0.66±0.13	0.64± 0.14	0.64±0.24
Zr	0.72±0.16	0.73±0.37	0.71 ±0.94	0.71±0.95	0.70±0.94	0.70 ±0.96	0.69± 0.93	0.69± 0.94	0.69± 0.00	0.69±0.04
			322.0079	741.651	2932.7775	9429.761	2847104	277.446	491.8794	670.9241
P value			0.001	0.001	0.001	0.001	0.001	0.001	0.001	0.001
